# Eszopiclone and Zolpidem Produce Opposite Effects on Hippocampal Ripple Density

**DOI:** 10.3389/fphar.2021.792148

**Published:** 2022-01-11

**Authors:** Logan A. Becker, Hector Penagos, Francisco J. Flores, Dara S. Manoach, Matthew A. Wilson, Carmen Varela

**Affiliations:** ^1^ Department of Neuroscience and Behavior, Stony Brook University, Stony Brook, NY, United States; ^2^ Department of Neuroscience, University of Texas at Austin, Austin, TX, United States; ^3^ Psychology Department, Florida Atlantic University, Boca Raton, FL, United States; ^4^ Picower Institute for Learning and Memory, Massachusetts Institute of Technology, Cambridge, MA, United States; ^5^ Center for Brains Minds and Machines, Massachusetts Institute of Technology, Boston, MA, United States; ^6^ Department of Anesthesia, Critical Care and Pain Medicine, Massachusetts General Hospital, Harvard Medical School, Boston, MA, United States; ^7^ Department of Psychiatry, Massachusetts General Hospital, Harvard Medical School, Boston, MA, United States; ^8^ Athinoula A. Martinos Center for Biomedical Imaging, Charlestown, MA, United States

**Keywords:** hippocampus, sharp-wave ripples, memory, eszopiclone, zolpidem, sleep, insomnia treatment

## Abstract

Clinical populations have memory deficits linked to sleep oscillations that can potentially be treated with sleep medications. Eszopiclone and zolpidem (two non-benzodiazepine hypnotics) both enhance sleep spindles. Zolpidem improved sleep-dependent memory consolidation in humans, but eszopiclone did not. These divergent results may reflect that the two drugs have different effects on hippocampal ripple oscillations, which correspond to the reactivation of neuronal ensembles that represent previous waking activity and contribute to memory consolidation. We used extracellular recordings in the CA1 region of rats and systemic dosing of eszopiclone and zolpidem to test the hypothesis that these two drugs differentially affect hippocampal ripples and spike activity. We report evidence that eszopiclone makes ripples sparser, while zolpidem increases ripple density. In addition, eszopiclone led to a drastic decrease in spike firing, both in putative pyramidal cells and interneurons, while zolpidem did not substantially alter spiking. These results provide an explanation of the different effects of eszopiclone and zolpidem on memory in human studies and suggest that sleep medications can be used to regulate hippocampal ripple oscillations, which are causally linked to sleep-dependent memory consolidation.

## Introduction

Noninvasive interventions of sleep oscillations can be used to normalize or enhance memory function (Malkani and Zee, 2020; Manoach et al., 2020; Winkelman and Lecea, 2020). Sleep-dependent memory consolidation relies on oscillations of non-rapid eye movement (NREM) sleep: hippocampal sharp-wave ripples, thalamocortical spindles, and cortical slow oscillations (Staresina et al., 2015; Maingret et al., 2016; Latchoumane et al., 2017; Klinzing et al., 2019; Ngo et al., 2020; Varela and Wilson, 2020). Ripples are particularly crucial because these brief, high-frequency oscillations (100–275 Hz), correspond to the reactivation of hippocampal neuronal ensembles that contribute to memory (Wilson and McNaughton, 1994; Girardeau et al., 2009; Ego-Stengel and Wilson, 2010). Therapeutics that enhance ripples could be valuable to treat memory deficits in several disorders.

Eszopiclone and zolpidem, two non-benzodiazepine sedative hypnotics that are prescribed to treat insomnia (Richey and Krystal, 2011; Matheson and Hainer, 2017; Liang et al., 2019; Krystal, 2020), are among the candidates to normalize sleep oscillations and memory (Kaestner et al., 2013). Zolpidem binds preferentially to GABA (A) receptors with the *α*1 subunit, while eszopiclone has a broad affinity (Hanson et al., 2008; Dixon et al., 2015); these differences in pharmacology can translate into differential effects at the cellular and network level (Jia et al., 2009). Eszopiclone increased spindles in humans but failed to enhance overnight memory consolidation (Wamsley et al., 2013; Mylonas et al., 2020). In contrast, recent findings suggest that zolpidem improves memory in humans (Niknazar et al., 2015; Zhang et al., 2020). Rodent studies looked at the effect of eszopiclone on cortical oscillations but not ripples (Xi and Chase, 2008; Hambrecht-Wiedbusch et al., 2010; Huang et al., 2010; Methippara et al., 2010; Fox et al., 2013), and there are few studies of zolpidem in rodents (Ponomarenko et al., 2004; Koniaris et al., 2011; Berdyyeva et al., 2014). We hypothesized that the contrasting effects of these drugs on memory consolidation were due to differential effects in hippocampal function.

Here we describe results from extracellular recordings in rats that suggest that eszopiclone disrupts neuronal spiking and ripple occurrence in the hippocampus, while zolpidem increases ripple occurrence. The results could contribute to the contrasting effects of eszopiclone and zolpidem in human studies.

## Materials and Methods

We investigated the effect of eszopiclone and zolpidem using microdrive arrays with tetrodes (Kloosterman et al., 2009; Nguyen et al., 2009) and an intraperitoneal tube for drug dosing chronically implanted in Long-Evans male rats (400–500 g; *n* = 4; purchased from Charles River Laboratories). The electrodes were lowered to the CA1 cell layer to record local field potentials (LFPs; sampled at 600 Hz) and spikes during natural sleep. Rats were housed individually, provided with food and water ad libitum, and monitored in a temperature-controlled room with a 12 h light/dark cycle (lights on/off at 7:00 am/7:00 pm). Experiments were approved by the Committee on Animal Care at the Massachusetts Institute of Technology and conform to US National Institutes of Health guidelines for the care and use of laboratory animals.

All rats received drug and vehicle infusions (*n* = 4 rats). Eszopiclone was tested in three rats and zolpidem in 2 (the sequence of injections per rat is included in Supplementary Table S2). In each session, rats were placed in a quiet area and their sleep and wakefulness were monitored through the LFP from CA1 and the retrosplenial cortex. Before injection, we recorded up to 1 hour to obtain baseline hippocampal activity; we injected eszopiclone (10 mg/kg), zolpidem (10 mg/kg) or vehicle and recorded the effect for 4–6 h. Dosing was performed between 11.00am and 12.30pm with the exception of one injection of zolpidem that was performed at 2.45pm but we did not find differences in its effect. For both drugs we selected a high i. p. dosage (10 mg/kg) based on dose-response rodent studies (Gauthier et al., 1997; Xi and Chase, 2008; Huang et al., 2010; Methippara et al., 2010). Drug injections were separated by at least 1 week to allow for drug clearance. Electrode location was confirmed in brain sections and with LFP signatures of cortex (delta and spindle oscillations) and hippocampus (ripples).

### Surgical Implantation

Aseptic surgery procedures were performed to implant tetrode arrays and an intraperitoneal (i.p.) polyurethane infusion catheter. The microdrive implant was secured to the skull with dental cement and bone screws. The infusion catheter (ID 0.027 in, OD 0.047 in; Harvard Apparatus) was implanted in the peritoneal cavity, and the excess tubing run subcutaneously to the back of the neck, where it was secured by dental cement and remained closed until dosing. Animals were preemptively injected with analgesics (Buprenorphine, 0.5–1 mg/kg, subcutaneous), and monitored for 3 days post-surgery.

### Drug Preparation

Eszopiclone and zolpidem were purchased from Sigma in powder form and prepared within 48 h of their application by dissolving in sodium acetate buffer 50 mM pH 4, filtered through a 0.22 µm filter; zolpidem was first dissolved in 5% DMSO, then mixed with the filtered sodium acetate buffer. The vehicle consisted of sodium acetate buffer 50 mM pH 4 (for eszopiclone) and sodium acetate buffer 50 mM pH 4 with DMSO (for zolpidem). The drugs were stored at 4°C; on the experimental day, the solution was drawn into a sterile syringe for immediate administration.

### Sleep Detection

We detected NREM sleep and ripples as described before (Ji and Wilson, 2007; Varela and Wilson, 2020). Briefly, we looked for periods of high cortical delta (1–4 Hz) and CA1 ripple (100–275 Hz) power in the LFP, or for periods of high cortical delta (ripple-independent sleep detection; Supplementary Figure S3C-D). Sleep periods of less than 120 s were disregarded, as were the 10 mins preceding and following injections to avoid interference from the procedure. Ripples were detected from the squared filtered (100–275 Hz) CA1 LFP as periods with power greater than 3 standard deviations above the mean ripple power for at least 20 ms.

### LFP and Spike Analyses

We analyzed both LFPs (which represent input into a brain region) and spikes, which represent the output from CA1 (Herreras, 2016). Spectrograms were calculated with a moving window of 20 s using the mtspecgramc function in the Chronux package, and power spectra using the pspectrum function in MATLAB. To obtain a metric of LFP ripple activity independent of ripple detection methods, we quantified changes in ripple power in the spectrograms relative to the baseline. We then detected ripples as described in previous work (Ji and Wilson, 2007; Varela and Wilson, 2020) and calculated ripple density as the number of ripples per second. Spike rates were calculated as spikes per second; to study spikes during ripples, we calculated peri-ripple histograms by averaging the spike counts (bin = 5 ms) in a 200 ms window around ripples. In most sessions, we normalized spike rates to the rate in the sleep before dosing. In three sessions with less than 15 min of sleep before injection, we normalized the firing rate using baseline recordings (session with no injection) from the same rat. We further normalized by the average rate within the baseline session and by its duration. This took into account variations in spike counts and session length in different days.

### Spike Waveform Classification

To separate spikes from putative pyramidal cells and interneurons, we used a threshold on the peak-to-trough waveform duration at 0.35 ms (Csicsvari et al., 1999a; Nowak et al., 2003; Le Van Quyen et al., 2008). Confirmation of spike type was done by examining the autocorrelation of spike times within each cluster and by comparing the resulting clusters to spikes sorted based on amplitude projections by an expert user.

### Statistics

Given the multi-variate nature of the data, which varies as a function of time, drug, and inter- and intra-rat conditions, we tested significance by fitting the data to a linear mixed model with session as the experimental unit and rat as a random effect, and calculated *p*-values through an F-test that compares the linear regression model coefficients. The model was computed using MATLAB’s function *fitlme* and the *p*-values were extracted using *coefTest*. Statistical results are reported for each analysis as the size of the effect (percent change), the statistical value, the degrees of freedom, and the *p* value; statistics are summarized in Supplementary Table S1.

## Results

### Decrease in CA1 Ripple Power After Eszopiclone and Increase After Zolpidem

**FIGURE 1:**
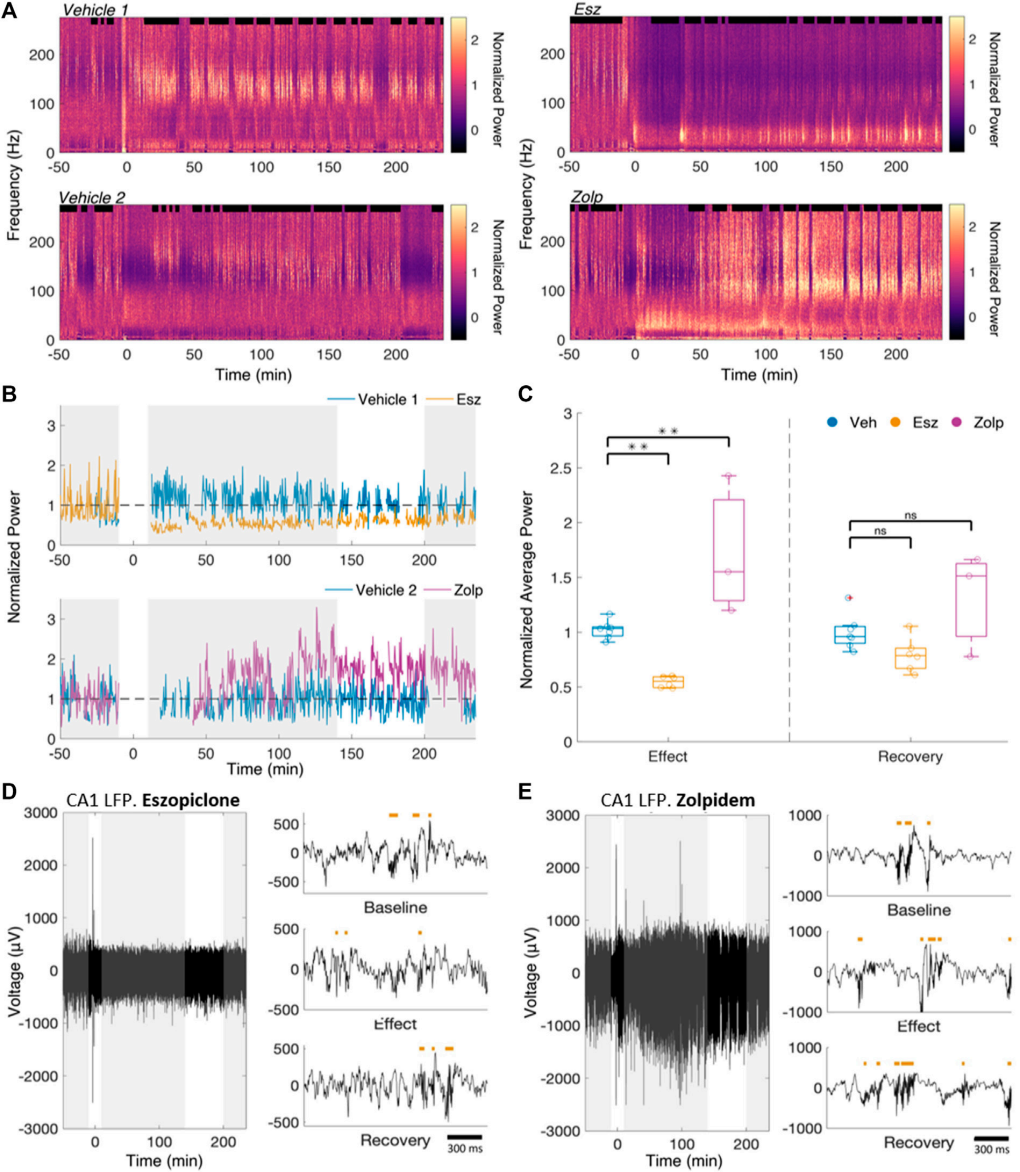
Eszopiclone and zolpidem have differential effects on CA1 power in the ripple band. **(A)** Raw spectrogram of the CA1 LFP for a pair of vehicle and eszopiclone sessions **(top)** and vehicle and zolpidem sessions **(bottom)** from the same rat. Drug (10 mg/kg) or vehicle was injected at time 0. **(B)** Average normalized ripple power (100–200 Hz) during sleep for the spectrograms in Figure 1A. Gray boxes indicate time windows of interest **(from left to right:** Baseline, Effect, Recovery**)**. Effects near the injection (*t* = 0) were ignored. **(C)** Boxplots (each circle one session) of the ratio of average normalized sleep ripple power (100–200 Hz), within “Effect” and “Recovery” relative to the “Baseline”. Significance was determined using an F-test on a fitted mixed linear model (*n* = 4 rats; see Supplementary Table S2 for details). **(D)** Example raw CA1 LFP recordings from a session in which eszopiclone was injected, along with detected ripples (red bars) within the three time windows of interest** (top,** Baseline; **middle,** Effect; **bottom,** Recovery). **(E)** Same for zolpidem session.

To assess the effect of the two drugs on ripples, we first calculated spectrograms of the CA1 LFP for drug and vehicle sessions (Figure 1A; power spectra and spectrograms for all drug sessions and sample vehicle sessions in Supplementary Figures S1,2). The spectral power after dosing was normalized to the average baseline sleep power for each frequency. Dosing was performed through the intraperitoneal cannula and is considered time 0 (t_0_) in all sessions.

We classified NREM as periods of high delta (1–4 Hz) power in cortex and high ripple power in CA1 (Ji and Wilson, 2007; Davidson et al., 2009; Varela and Wilson, 2020). We saw no significant difference in the average sleep bout duration between vehicle, eszopiclone or zolpidem sessions (10.98, 12.46 and 11.55 min respectively; vehicle/eszopiclone, F (1,13) = 1.46; *p* = 0.25; vehicle/zolpidem, F (1,13) = 0.26, *p* = 0.62), nor in the average number of bouts per hour (2.92 bouts/hour, 2.93 bouts/hour and 3.01 bouts/hour respectively; vehicle/eszopiclone, F (1,13) = 1.41; *p* = 0.26; vehicle/zolpidem, F (1,13) = 1.96, *p* = 0.19; Supplementary Figure S3A). We also obtained no significant drug effects on sleep time (vehicle/eszopiclone, F (1,13) = 0.89; *p* = 0.36; vehicle/zolpidem, F (1,13) = 0.09, *p* = 0.77) or sleep bouts (vehicle/eszopiclone, F (1,13) = 0.06; *p* = 0.81; vehicle/zolpidem, F (1,13) = 0.002, *p* = 0.96) with a classification of NREM based only on cortical delta power (thus independent of CA1 ripple power; Supplementary Figure S3B). However, we observed a drop in ripple power (Figure 1B) following the injection of eszopiclone*,* and an increase following zolpidem.

We defined three time windows based on their pharmacokinetics in rats (Gaillot et al., 1983; Trenque et al., 1994): *baseline* (50–10 min pre-injection), *effect* (10–140 min post-injection), and *recovery* (200–235 min post-injection). We took the ratio of the sleep ripple power during *effect* and *recovery* to the *baseline* power (normalized as described in Methods; Figure 1C). If there were less than 15 min of detected sleep within the baseline (3 sessions), we instead used the first hour of a different recording session from the same rat in which there was no injection. During the effect, eszopiclone decreased ripple power by 44%, whereas zolpidem increased it by 97% (vehicle/eszopiclone, F (1,13) = 11.11; *p* = 0.005; vehicle/zolpidem, F (1,13) = 15.83, *p* = 0.002; Supplementary Table S1). The normalized sleep ripple power during recovery was not significantly different compared to vehicle (19% below baseline in eszopiclone and 50% above baseline with zolpidem; vehicle/eszopiclone, F (1,13) = 2.53; *p* = 0.14; vehicle/zolpidem, F (1,13) = 3.77, *p* = 0.07). These results suggest that eszopiclone has an acute adverse effect on ripple power, while zolpidem increases hippocampus ripple activity.

### Eszopiclone Decreases and Zolpidem Increases CA1 Ripple Density

**FIGURE 2:**
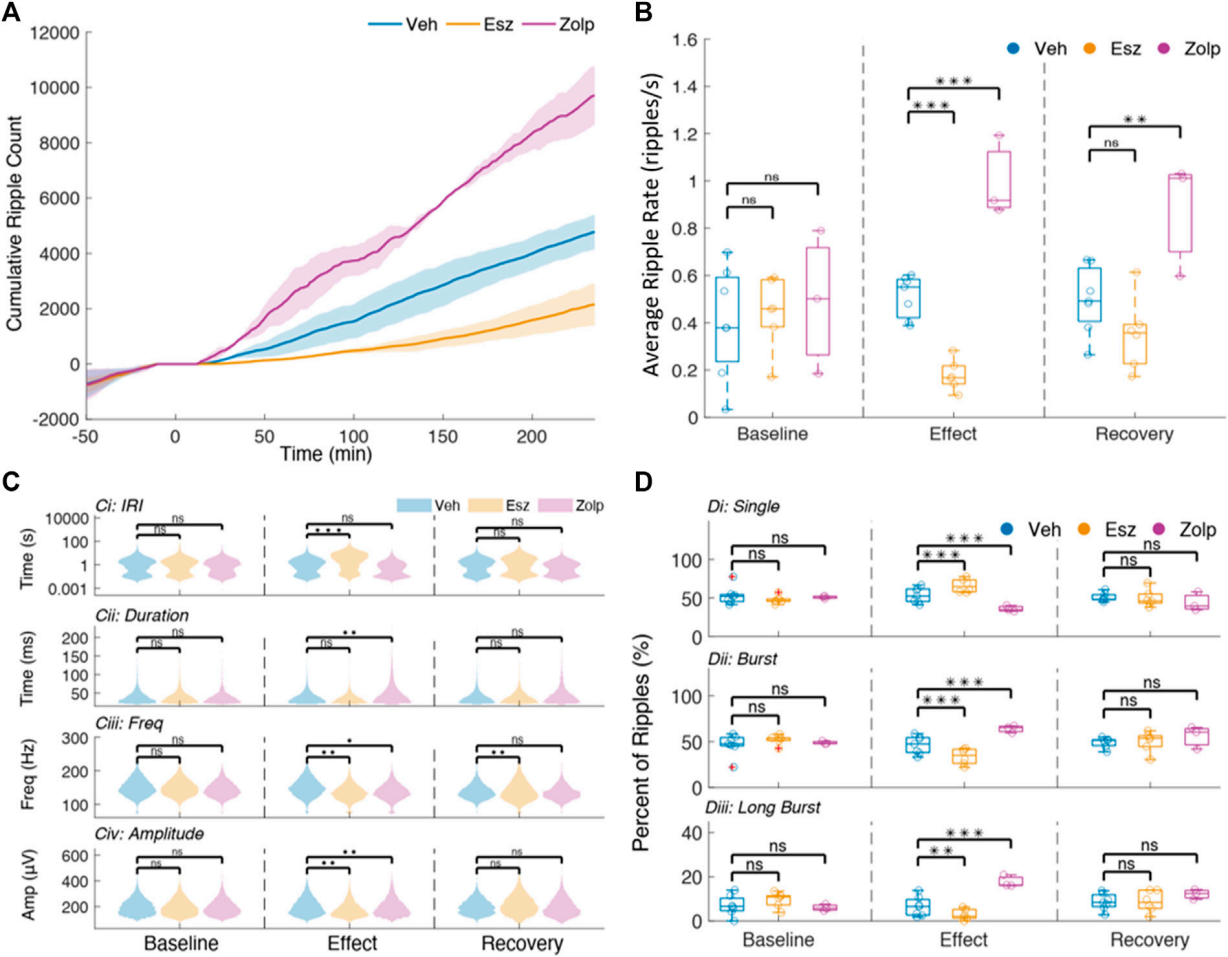
Ripple sparsity increases after eszopiclone and decreases with zolpidem. **(A)** Mean cumulative ripple counts across sessions with shaded standard deviations for vehicle, Eszopiclone and Zolpidem (*n* = 4 rats; see Supplementary Table S2 for details). Time 0 indicates injection. **(B)** Mean ripple rate (per second) within each time window of interest in all sessions (each circle one session). Significance was determined using an F-test on a fitted mixed linear model. **(C)** Violin plots of Inter-Ripple-Intervals **(top row,** *y*-axis log scaled**)**, Ripple duration (second row), Intra-ripple frequency (third row) and normalized ripple amplitude **(bottom row)**, comparing Vehicle (blue), Esz (red) and Zolp (purple) across time windows: Baseline **(left)**, Effect **(middle),** and Recovery **(right)**. **(D)** Distribution of the proportion of “single” ripples (Di), ripple bursts (Dii) and “long” ripple bursts (bursts with four ripples or more; Diii) for all sessions during Baseline, Effect and Recovery.

Noting a change in ripple power, we analyzed whether eszopiclone and zolpidem modulate the number of ripples (Figures 1D,E). The cumulative ripple count showed that eszopiclone decreased, while zolpidem increased, the number of ripples (Figure 2A). Specifically, the ripple density (ripples per second) significantly decreased after eszopiclone (by an average of 60%; *n* = 3 rats) and increased after zolpidem [by 102%; *n* = 2 rats; vehicle/eszopiclone, F (1,13) = 43.66; *p* = 1.7e-5; vehicle/zolpidem, F (1,13) = 52.46, *p* = 6.5e-6; Figure 2B]. To resolve the time course of the effect, we looked at the ripple density every 30 min and found that eszopiclone reached a maximum effect within 30 min of injection while the increase in ripples induced by zolpidem had slower dynamics (Supplementary Figure S4). With both drugs, the ripple density did not fully recover to baseline levels within the recorded period (up to 6 h), suggesting that they can have a prolonged effect on hippocampal ripples.

The generation of ripples relies on interactions between hippocampus sub-regions (Buzsáki et al., 1983; Carr et al., 2012; Csicsvari et al., 1999b; Yamamoto and Tonegawa, 2017), while the morphology of the ripple oscillation depends on interactions between CA1 pyramidal cells and interneurons (Klausberger and Somogyi, 2008; Stark et al., 2014; Ylinen et al., 1995). To gain insight on the mechanisms by which eszopiclone and zolpidem affect ripples, we studied several ripple features: inter-ripple-interval, duration, intrinsic ripple frequency and peak-to-trough amplitude (Figure 2C; Supplementary Table S1). Ripples often occur in bursts (Davidson et al., 2009; Yamamoto and Tonegawa, 2017), and we also quantified the effect of the drugs on ripple bursts (Figure 2D).

The baseline inter-ripple interval (IRI) distributions were bimodal (Figure 2C), with the first peak indicating ripple bursts, while the second implies ‘single’ events. Eszopiclone shifted the mean to longer IRIs (from an average 2°s IRI in baseline to 6°s during the effect) thus increasing ripple sparsity, while zolpidem shifted the mean to shorter IRIs (from 2 to 1°s). These shifts do not by themselves entail a change in the proportion of ripple bursts and single ripples; to estimate that, we calculated the percent of “single” ripples and ripples in bursts (based on the IRI at the minimum between the two modes for each session). In eszopiclone, ripple bursts dropped significantly to 34% (compared to 52% during Baseline) and the fraction of single ripples increased proportionally. Zolpidem increased ripple bursts to 64% (from 49% in Baseline). To confirm the effect, we used a more stringent classification of bursts by detecting “long” bursts (4 ripples or more) and again found a significant decrease of long bursts with eszopiclone and a significant increase with zolpidem. These results (Figure 2D; Supplementary Table S1) suggest that the two drugs change ripple density by disrupting (eszopiclone) or enhancing (zolpidem) the grouping of ripples [vehicle/eszopiclone, F (1,13) = 33.84; p = 6e-5; vehicle/zolpidem, F (1,13) = 1.42; *p* = 0.26].

In addition, we found a slight but significant decrease in intra-ripple frequency with eszopiclone [10% during effect; F (1,13) = 16.95; *p* = 0.001, and 8% during recovery; F (1,13) = 16.68; *p* = 0.001] and a slight increase in intra-ripple frequency during the effect of zolpidem [1%; F (1,13) = 5.4; *p* = 0.04]. We also found that ripple duration increased during zolpidem [31%; F (1,13) = 15.45; *p* = 0.002], and saw a significant decrease [11% decrease; F (1,13) = 14.58; *p* = 0.002] in peak-to-trough amplitude with eszopiclone and a significant increase with zolpidem [8%, F (1,13) = 10.89; *p* = 0.01].

These results suggest that eszopiclone and zolpidem mainly affect the density of hippocampal ripples, with slight effects on the frequency, duration, and amplitude of the ripple oscillation. Both drugs had a strong but opposite effect on the density of ripple bursts, eszopiclone made ripples sparser while zolpidem made ripples denser.

### Decrease of Spike Activity in CA1 With Eszopiclone and Moderate Change Following Zolpidem

**FIGURE 3:**
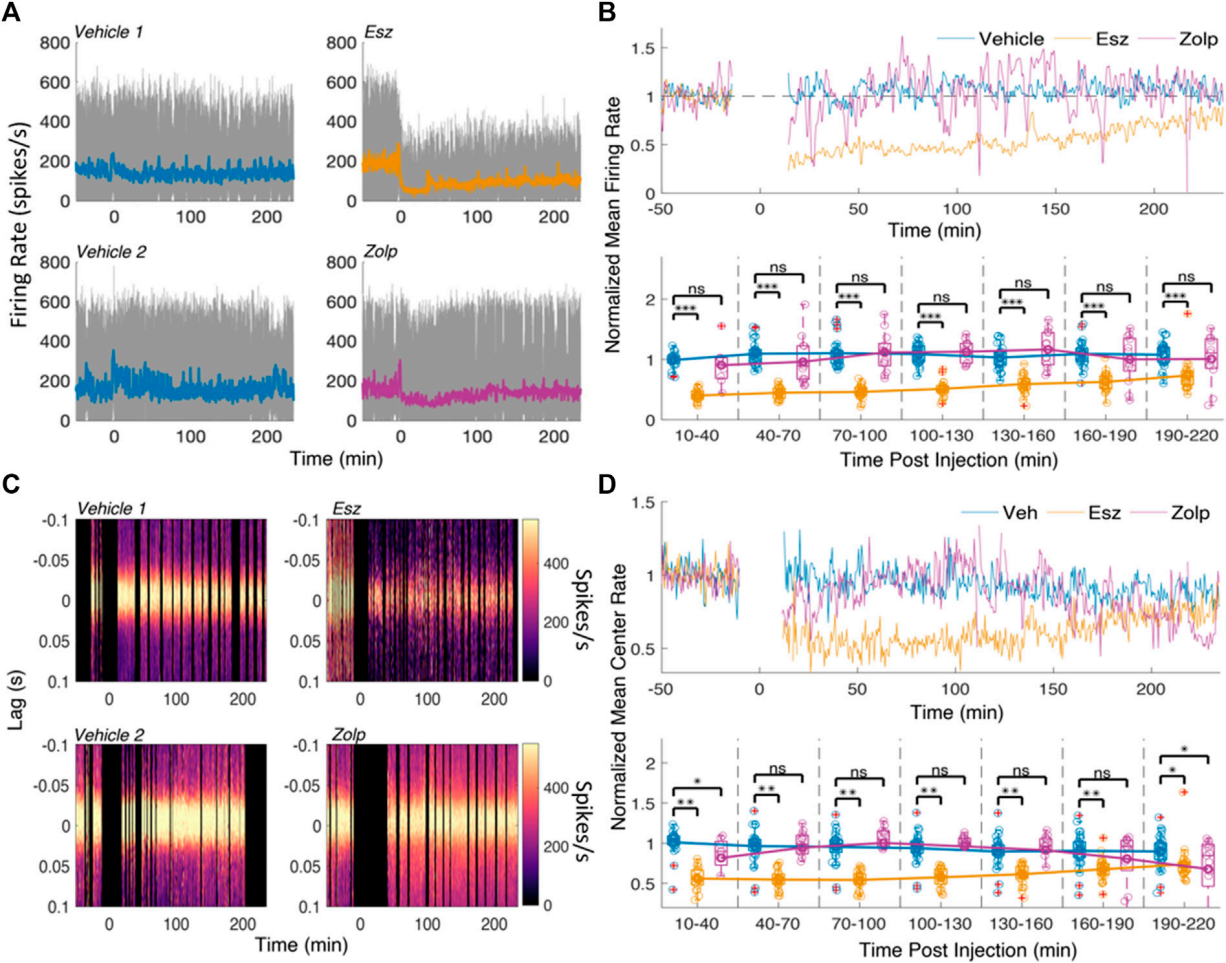
Spike rate decreases following eszopiclone and does not change significantly with zolpidem. **(A)** Multi-unit (MUA) firing rates for a pair of vehicle and eszopiclone sessions **(top)**, and a pair of vehicle and zolpidem sessions **(bottom)** from the same rat. Color line shows the mean rate over time. **(B)** Top: average firing rate across all sessions (*n* = 4; see Supplementary Table S2 for details), normalized by the mean baseline activity; spiking near injection (*t* = 0) was ignored. Bottom: Boxplots of the normalized mean rates in 30-minute time intervals post-injection with line connecting the means of each drug type (each circle corresponds to the spike rate in one CA1 electrode; red crosses indicate outliers). Significance was determined using an F-test on a fitted mixed linear model. **(C)** Examples of peri-ripple histograms of the MUA spikes over time for a pair of vehicle and eszopiclone sessions **(top)** and a pair of vehicle and zolpidem sessions **(bottom)**. Lag 0 corresponds to ripple events and color scheme shows the spike rate. Black vertical areas indicate periods with no sleep and the 10 minutes around injection. **(D)** Top: Mean MUA spike rate at the center (40 ms around lag 0) of the peri-ripple histograms, normalized by the average baseline activity, across all sessions; bottom: boxplots showing individual sessions in 30 min bins post-injection with lines connecting the means (each circle one tetrode). Significance was determined using an F-test on a fitted mixed linear model.

We next examined the effect on multi-unit spikes within the hippocampus (MUA, all spikes recorded by each tetrode; Figure 3A). We normalized the firing rate in each tetrode (4 CA1 tetrodes per session on average) to the average rate during baseline sleep. If there were less than 15 min of sleep within baseline, as before, we normalized using the first 60 min of sleep within a recording session with no injection. We looked at spikes in the three windows of interest (*baseline, effect* and *recovery*; results for all tetrodes in Supplementary Figure S5), and at the average spike activity every 30 min post-injection. Figure 3B shows the mean normalized firing rate in CA1 for all tetrodes (top) and broken down into 30 min intervals (bottom). Eszopiclone produced the highest drop in firing rate 10–40 min after injection [60%; F (1,47) = 137.22; *p* = 1.53e-15], followed by a slow recovery to baseline (27% decrease by 190–200 min; not different from vehicle). Zolpidem did not produce significant changes in spiking.

Although zolpidem did not change the overall firing rate, it is possible that spike firing is affected during ripples. To find out, we analyzed the effect of eszopiclone and zolpidem on the peak (40 ms around lag 0) of peri-ripple histograms of MUA. Figure 3C shows the peri-ripple histograms for the sessions in Figure 3A, and Figure 3D shows average (top) and binned (bottom) spike rates at the peak (40 ms around lag 0) of the peri-ripple histogram across all sessions.

Eszopiclone decreased ripple-associated spike rate through the session [45% 10–40 min post-injection; F (1,47) = 61.317; *p* = 4.57e-10]; zolpidem produced a small drop in ripple-associated spike rate [17% 10–40 min post-injection; F (1,47) = 6.51; *p* = 0.01], which was significant in the first and last 30 min bins. These results demonstrate that eszopiclone strongly decreases CA1 firing. The smaller drop with zolpidem, at the start and end of the recording, may reflect a dose-dependent effect on spike firing.

We tested one further possibility, which is that the drug effects could be specific to neuronal type. For example, zolpidem could enhance GABA currents in inhibitory interneurons (Gao and Fritschy, 1994; Klausberger et al., 2002; Baude et al., 2007), and disinhibit excitatory pyramidal cells; such an effect could explain the small changes in MUA, which includes both neurons. Instead, eszopiclone may affect both excitatory and inhibitory neurons due to its broad affinity for different GABA (A) receptors (Hanson et al., 2008). A spike width less than 350 μs was used to identify putative interneuron spikes (pInt), otherwise the waveform was classified as a putative pyramidal cell (pPy). Manual spike sorting and autocorrelograms similar to published data (Le Van Quyen et al., 2008) were used to validate the classification (Supplementary Figure S6).

**FIGURE 4:**
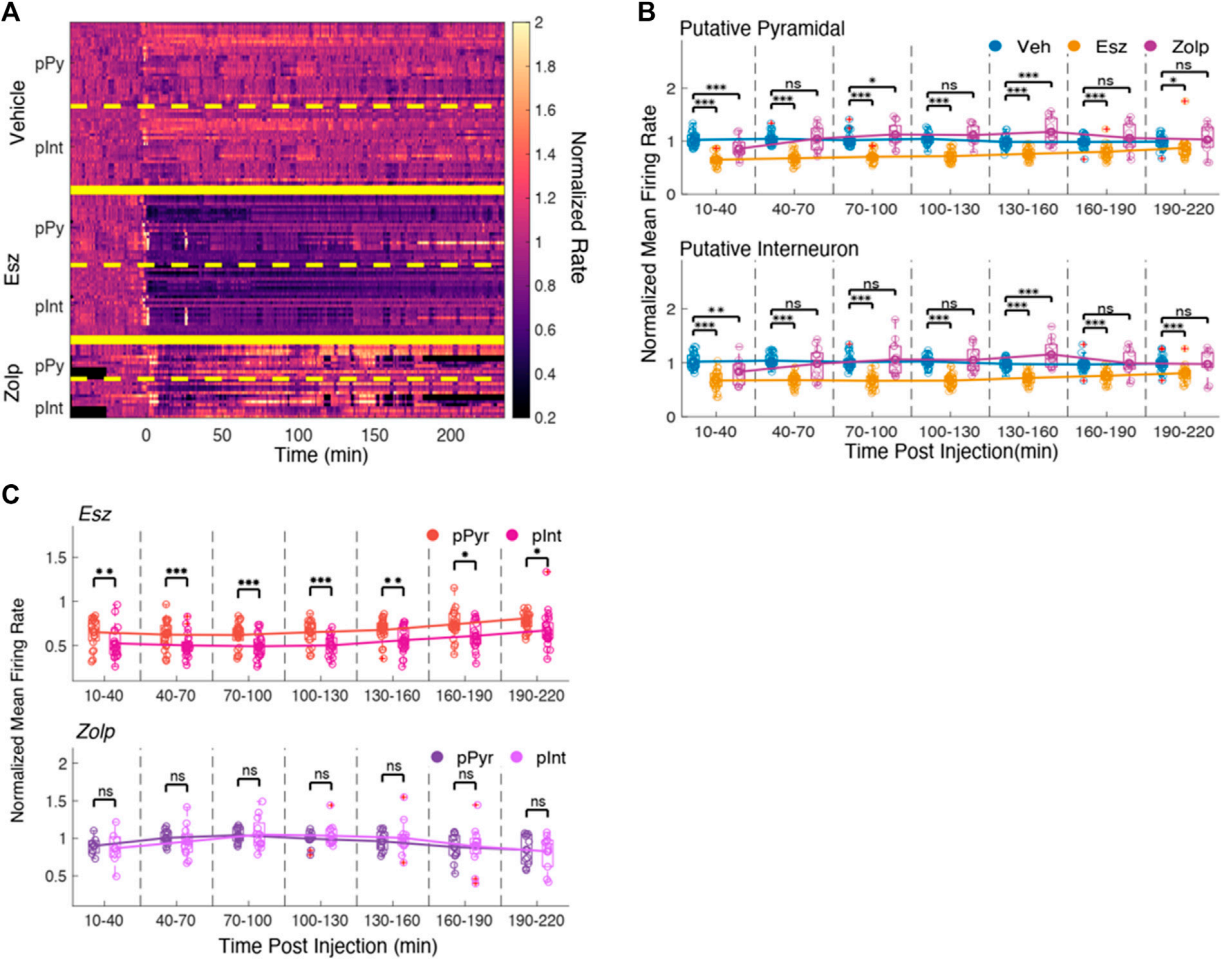
Eszopiclone and zolpidem have similar effects on spikes from putative pyramidal cells and interneurons. **(A)** Smoothed histograms of spike rate normalized by average rate during the ‘baseline’ time period for putative pyramidal cell spikes (pPy) and putative interneuron spikes (pInt). Rate matrix is divided into vehicle **(top)**, eszopiclone **(middle)** and zolpidem **(bottom)**, and further separated into putative pyramidal cells and putative interneurons (*n* = 4 rats). **(B)** Boxplots of the normalized mean spike rates for putative pyramidal cell **(top)** and putative interneuron spikes **(bottom)** in 30-minute time intervals post-injection with line connecting the means of each drug type. Each circle one tetrode; red crosses indicate outliers. Significance was determined using an F-test on a fitted mixed linear model. **(C)** Boxplots comparing side by side the effect on the peri-ripple histograms for putative pyramidal and putative interneuron spikes in eszopiclone **(top)** and zolpidem sessions **(bottom)**. Peri-ripple rates (normalized to baseline) are binned every 30 min post-injection with lines connecting the means. Significance was determined using an F-test on a fitted mixed linear model.

We examined the effect of the drugs on the two spike types by looking at their normalized average firing rate over time (Figure 4A). Eszopiclone produced a stark decrease in spike rate in both putative pyramidal cells and interneurons; with zolpidem we saw an initial decrease that increased and overshot the baseline rate, then recovered to near baseline. We saw no difference between the pyramidal and interneuron waveforms in vehicle, eszopiclone or zolpidem (Figure 4B). The strongest effect was with eszopiclone [pPy: 35%; F (1,63) = 98.41; *p* = 1.7e-14, pInt: 21%; F (1,63) = 53.91; *p* = 5.05e-10, during the first 10–30 min] and the effect was about half the size with zolpidem [pPy: 14%; F (1,63) = 13.1; *p* = 5.9e-4, pInt: 16%; F (1,63) = 10.43; *p* = 0.002].

### Stronger Disruption of Interneuron Ripple Correlations

Although we observed similar effects on the firing rates of putative pyramidal cells and interneurons, these two types of neurons contribute differently to ripples (Klausberger and Somogyi, 2008), and it is possible that their participation in ripples is differentially affected by eszopiclone or zolpidem. We therefore compared the spike rate at the center of the peri-ripple histogram for the putative pyramidal cells and interneurons in eszopiclone and zolpidem (Figure 4C). Only after eszopiclone the peri-ripple histogram of interneuron spikes was significantly lower relative to the peri-ripple histogram of putative pyramidal neurons [10–30 min: F (1,36) = 11.04; *p* = 0.002, 130–160 min: F (1,48) = 11.01; *p* = 0.002, 190–220 min: F (1,48) = 4.85; *p* = 0.03], indicating that eszopiclone may produce a stronger disruption of the timing between ripples and interneuron spikes.

## Discussion

Eszopiclone and zolpidem produced opposite effects on sleep-dependent memory consolidation in humans. To gain mechanistic insight on how these drugs could produce opposing effects on memory, we investigated their effect on ripples and spikes in rat CA1 during sleep. We found that eszopiclone significantly decreased ripple density while zolpidem significantly increased ripple density and their grouping in bursts. The drugs had small effects on the ripple oscillation: both drugs reduced intra-ripple frequency slightly, and zolpidem increased ripple duration; ripple amplitude was smaller after eszopiclone and larger after zolpidem. In addition, eszopiclone strongly reduced spiking in putative pyramidal cells and interneurons, whereas zolpidem had small effects on CA1 spikes. The different effects on ripples and cell firing may help explain why zolpidem (but not eszopiclone) had beneficial effects on memory consolidation in human studies.

### Pharmacological Regulation of Ripple Density

Eszopiclone and zolpidem changed ripple density without substantial changes to the ripple waveform. Both drugs affected primarily ripple bursts, suggesting an effect on the circuits that initiate and group ripples (CA3; entorhinal cortex -EC-). Three mechanisms could explain the effects on ripple density: changes in CA3 neuron excitability, which produced increases and decreases in ripple density (Schlingloff et al., 2014). Modulation of the pathway from medial EC to CA1, which correlated with greater incidence of ripple bursts (Yamamoto and Tonegawa, 2017). Lastly, ripple density was associated with neuronal activity in the raphe (Logothetis et al., 2012; Wang et al., 2015), which could be affected by activation of GABA (A) receptors.

Eszopiclone has a broad GABA (A) receptor affinity (Hanson et al., 2008) and could produce a general enhancement of GABA (A) currents and decrease spike activity directly in CA1, or indirectly by increasing GABA (A) currents in neuromodulatory centers (Hasselmo and McGaughy, 2004; Olsen and Sieghart, 2009; Dixon et al., 2015). Zolpidem binds preferentially to GABA (A) receptors with *α*1 subunits (Hanson et al., 2008; Fitzgerald et al., 2014; Che Has et al., 2016), which mediate inhibition from parvalbumin interneurons (Gao and Fritschy, 1994; Klausberger et al., 2002; Bacci et al., 2003; Baude et al., 2007). A more restricted cell population target could explain the subtler effects on spike rates we observed with zolpidem, which are consistent with mild effects of zolpidem on hippocampal theta (Yoshimoto et al., 1999).

Although our sample size was small, the results were consistently observed in all sessions and animals, and are supported by a linear mixed-effects models to treat sessions as experimental units and rats as random effects. Future dose-response studies will be important to make sense of the different zolpidem effects among hippocampus studies. Zolpidem increased ripples in hippocampal slices (Koniaris et al., 2011), but a study in Wistar rats found a transient reduction in ripples (Ponomarenko et al., 2004); likewise, work in mice (Berdyyeva et al., 2014) found a decrease in calcium transients (which may correspond to ripples and to subthreshold activity) in CA1 after oral zolpidem dosing. The variability across studies suggest that the effect of zolpidem on hippocampus activity depends on the bioavailability and effective dosage achieved in different preparations. In this respect, we found that zolpidem transiently decreased ripple-associated spikes after injection and in the last 30 min of recording, which may reflect changes in its brain concentration.

### Drug Interventions to Regulate Sleep Oscillations and Cognition

In humans, eszopiclone failed to restore sleep-dependent memory consolidation in either healthy people or schizophrenia patients (Wamsley et al., 2013; Mylonas et al., 2020). In contrast, zolpidem enhanced sleep-dependent memory consolidation (Kaestner et al., 2013; Mednick et al., 2013; Niknazar et al., 2015; Zhang et al., 2020). Pharmacology studies of the effect of eszopiclone and zolpidem on memory are more limited in the animal literature. Eszopiclone and zolpidem reduced freezing behavior after contextual fear training in rats, consistent with a disruption of memory consolidation, although reduced freezing may also result from anxiolytic effects (Huang et al., 2010). Rodent work suggests a direct link between ripples, memory reactivation and consolidation (Foster, 2017; Joo and Frank, 2018; Klinzing et al., 2019). Invasive techniques that disrupt or enhance ripples produced, respectively, memory impairment or enhancement (Girardeau et al., 2009; Ego-Stengel and Wilson, 2010; Fernández-Ruiz et al., 2019). A further step to understand the mechanisms of action of eszopiclone and zolpidem will be to determine if the changes in ripple rate translate into opposite effects on memory. The decrease of spike activity with eszopiclone may contribute to additional effects on behavior. Another important aspect will be to investigate the effect of both drugs on the coupling of ripples with spindle and slow oscillations, which is thought to play a crucial role in systems memory consolidation (McClelland et al., 1995; Staresina et al., 2015; Varela and Wilson, 2020).

## Data Availability

The raw data supporting the conclusion of this article will be made available by the authors, without undue reservation.
