# Supplementary material for: Eszopiclone and Zolpidem Produce Opposite Effects on Hippocampal Ripple Density
Source: Front Pharmacol. 2022 Jan 11;12:792148. doi: 10.3389/fphar.2021.792148 (PMC8787044; doi:10.3389/fphar.2021.792148)
Supplement: Supplementary file 1 [file DataSheet1.docx]

**Supplementary Table 1.** **Statistical results.** Summary of effect size and p-values for ripple features and spike analysis during the effect window (10-40 min after injection of eszopiclone or zolpidem; exact p-values provided in text when < 0.001).

|  | **Eszopiclone** | | **Zolpidem** | |
| --- | --- | --- | --- | --- |
|  | *% change* | *P value (F test)* | *% change* | *P value (F test)* |
| **Ripple Features** |  |  |  |  |
| Ripple power | -44 | 0.005 | 97 | 0.002 |
| Density per second | -60 | < 0.001 | 102 | < 0.001 |
| Inter-ripple-interval | 142 | < 0.001 | -38 | 0.255 |
| Peak-to-trough | -11 | 0.002 | 8 | 0.006 |
| Frequency | -10 | 0.001 | 1 | 0.037 |
| Duration | -12 | 0.128 | 31 | 0.002 |
| % ripple bursts | -35 | < 0.001 | 31 | < 0.001 |
| % long ripple bursts (≥ 4 ripples) | -70 | < 0.001 | 200 | < 0.001 |
| **Spike Analysis** |  |  |  |  |
| MUA spike rate | -60 | < 0.001 | -7 | 0.786 |
| Peri-ripple peak MUA firing | -45 | < 0.001 | -17 | 0.014 |
| Putative pyramidal cell spike rate | -35 | < 0.001 | -14 | < 0.001 |
| Putative interneuron spike rate | -21 | < 0.001 | -16 | 0.002 |

**Supplementary Table 2.** **Sequence of Experimental Sessions with Drug/Vehicle Injections for all rats.** ESZ = eszopiclone injection; ZOLP = zolpidem injection; VEH = vehicle injection.

| **RAT** | **Session Order** |
| --- | --- |
| 1 | ESZ; VEH; VEH; ESZ; ZOLP; VEH |
| 2 | ESZ; VEH; VEH; ESZ |
| 3 | ESZ; ESZ; VEH |
| 4 | ZOLP; VEH; ZOLP |

**Supplementary Figures (S1-6).**

Figure Captions.

**Supplementary Figure 1 (S1). Power spectra of CA1 LFP during ‘*baseline*’ (10-50mins prior to injection) and ‘*effect*’ (10-140mins after injection) for all eszopiclone and zolpidem injection sessions.** A) Eszopiclone sessions (n = 6); thicker line indicates the mean across sessions. B) Same for all zolpidem sessions (n = 3). Zoomed-in insets show the lower frequency bands (0-20 Hz). Line noise was removed using the Chronux toolbox.

**Supplementary Figure 2 (S2). Broad band spectrograms of CA1 LFP for drug and vehicle sessions.** A) CA1 spectrogram for all eszopiclone sessions and all rats (n = 3 rats; see Supplementary Table 2 for details); power before injection (time 0) was subtracted for each frequency band to reveal changes in power after injection. Horizontal black bars indicate detected sleep periods. B) Same for all zolpidem sessions (n = 2 rats). C) Examples of the CA1 LFP spectrogram and sleep periods in one vehicle session for each rat.

**Supplementary Figure 3 (S3).** **No difference of sleep patterns between Vehicle, Eszopiclone and Zolpidem sessions.** A) Average bout duration (detected based on ripple and delta power) during vehicle, eszopiclone, and zolpidem sessions. Right: Average bouts per hour in the same sessions. B) Same for sleep detection based only on delta power (ripple-independent sleep detection). C) Example of sleep detection methods in a vehicle injection session from one rat. Detected sleep bouts are indicated by horizontal lines on the plots of the average CA1 ripple power (top, black) and average cortical delta power (bottom, green) used for the detection; red horizontal lines indicate bouts detected based on both ripple and delta power being above the mean for the session; black horizontal lines indicate bouts detected only based on delta power being above the mean for the session; green dot indicates start of a sleep bout, red indicates the end; vertical blue line indicates the time of injection. D) Low-frequency (0-20 Hz) spectrogram of the cortical LFP for all eszopiclone sessions and all rats; detected sleep periods are marked by horizontal black bars (n = 3 rats; see Supplementary Table 2 for details); 0 indicates injection time. Horizontal black bars indicate detected sleep periods.

**Supplementary Figure 4 (S4).** **Ripple rates binned at 30 min intervals.** Mean ripple rate (ripples per second) before injection and every 30 minutes post-injection. Means are connected through solid colored line. Each data point consists of the average ripple rate within the given time interval for a given session (n = 4 rats; see Supplementary Table 2 for details).

**Supplementary Figure 5 (S5).** **Multi-unit (MUA) spike rates during Baseline, Effect and Recovery.** Boxplots of the normalized mean rates in all tetrodes within baseline (50-10 mins pre-injection), effect (10-140 mins post-injection) and recovery (200-235 mins post-injection). Each data point corresponds to one tetrode, red starts indicate outliers. Significance was determined using an F-test on a fitted mixed linear model.

**Supplementary Figure 6 (S6).** **Example of waveform classification to distinguish putative interneuron (pInt) from pyramidal cell (pPy) spikes.** A) Left: Histogram of spike width defined as the time from peak to trough in all waveforms recorded by one tetrode in a sample session. Right: Same histogram color coded based on a threshold of a peak-to-trough width of 350 μs. B) Extracted waveforms from the clustered data. Red line shows mean putative pyramidal cell spike waveform along with a sample of 1000 individual waveforms in light red. Black line shows mean putative interneuron trace along with a sample of 1000 individual waveforms in grey. Some putative interneurons can be further clustered by amplitude (dark gray and light gray traces). For the purpose of this work, the two clusters of putative interneurons were treated as one. C) Auto-correlation of putative pyramidal cell and interneuron spikes agrees with previous literature showing that pyramidal cells have a sharper exponential decay, compared to the more gradual shape of interneuron autocorrelations (Quyen et al., 2008). D) Distribution of spike width for clustered putative interneurons based on width threshold (light grey in panel S6b) compared to ground truth interneurons sorted manually based on spike amplitude projections.

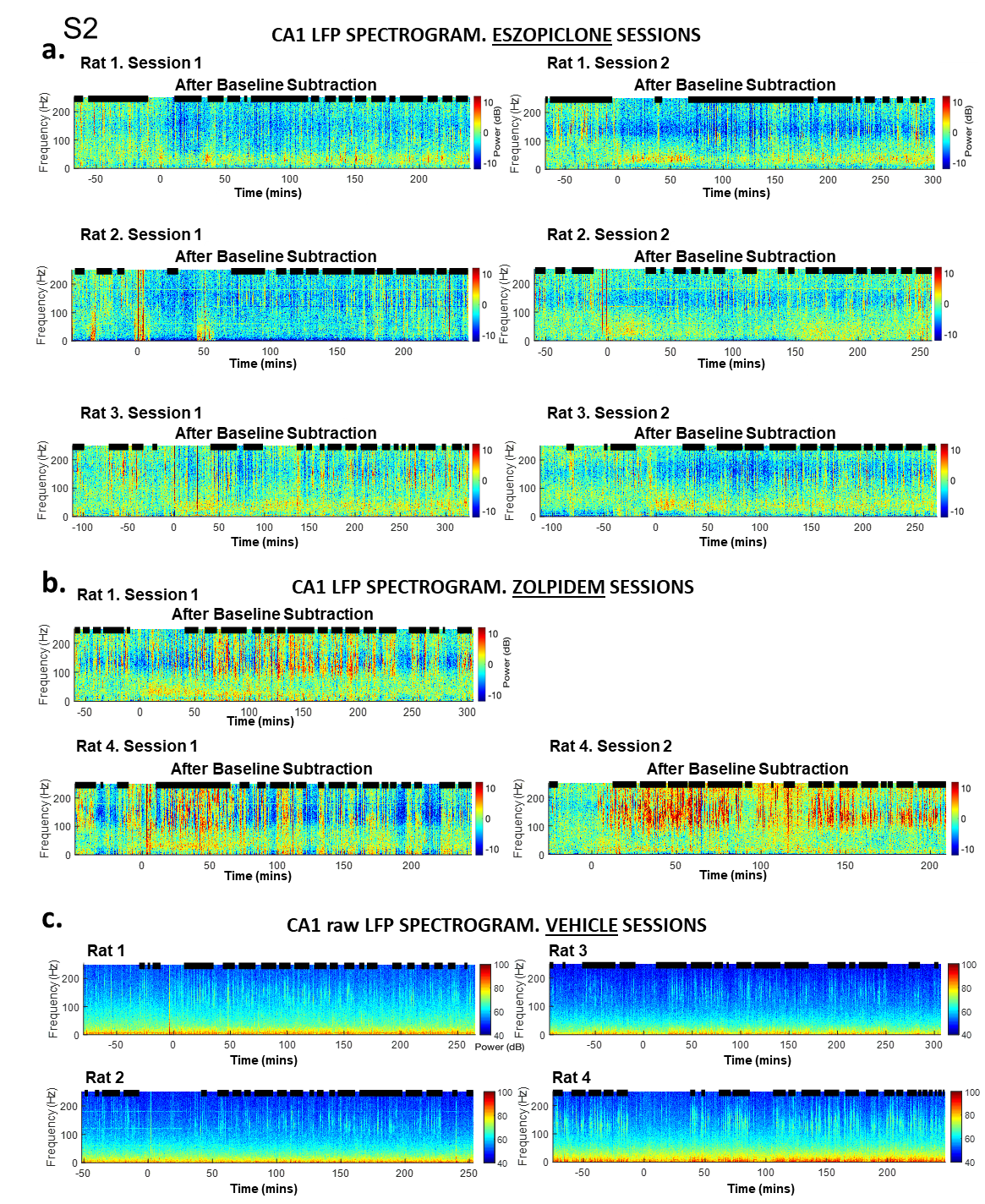


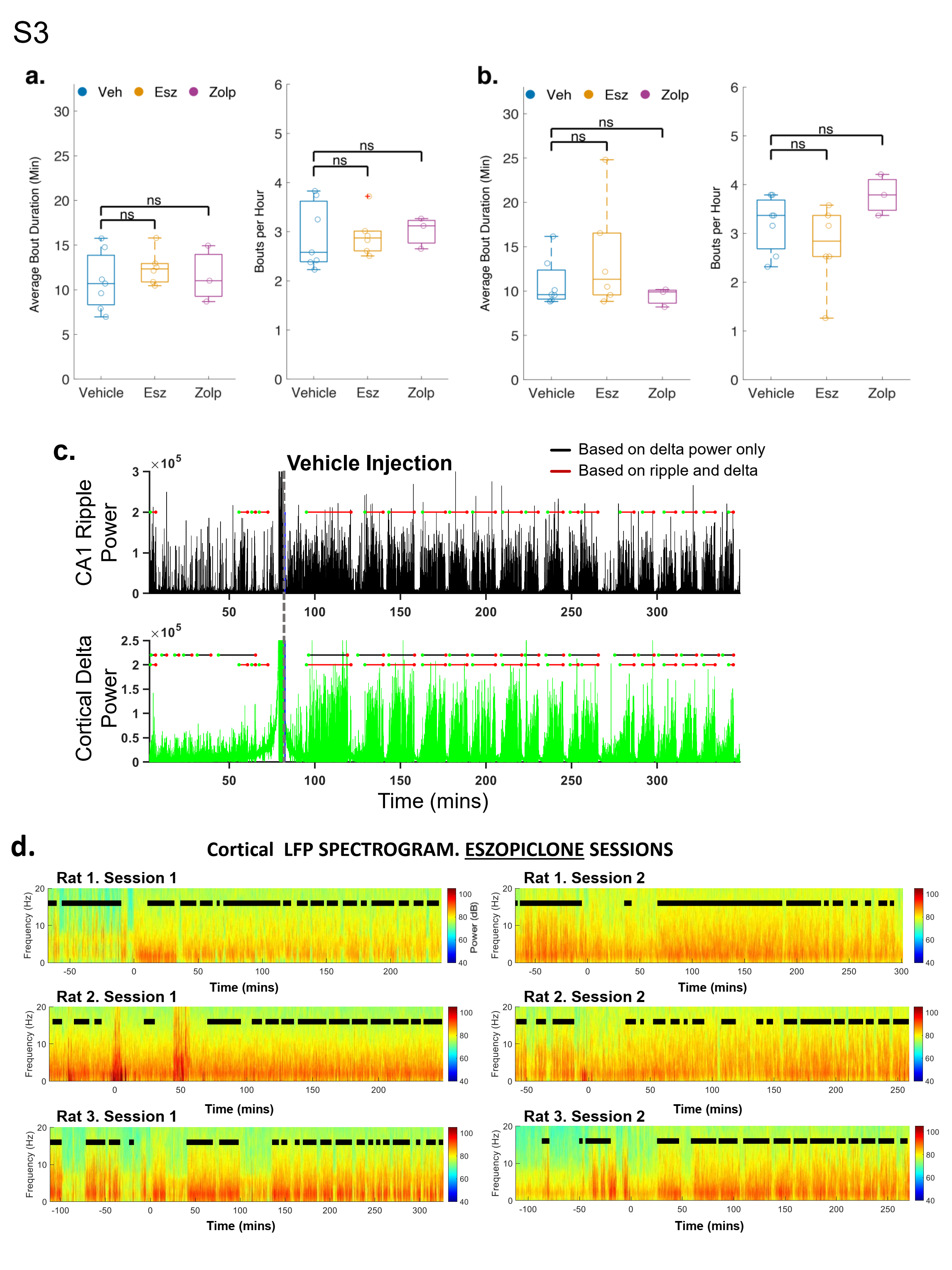


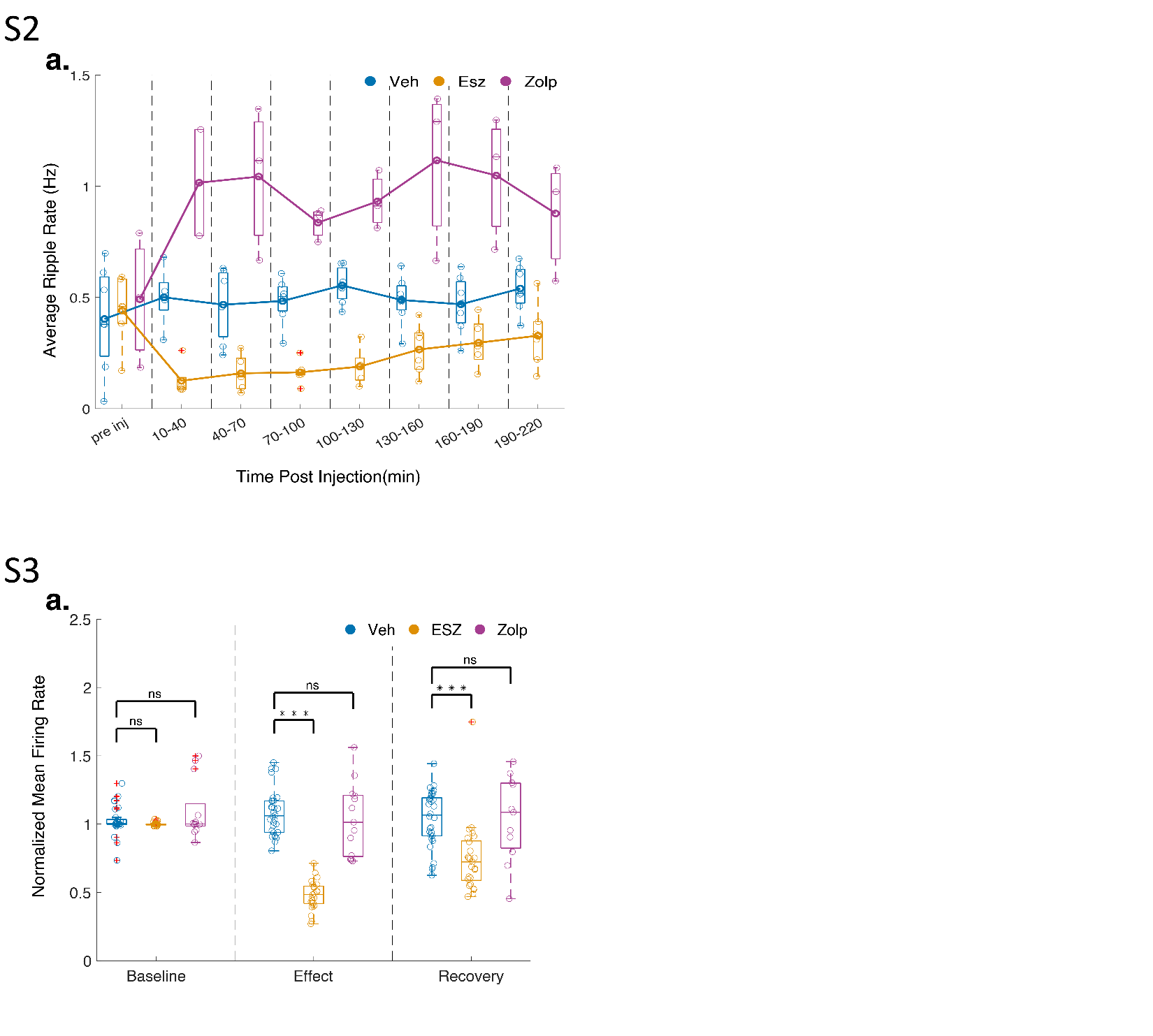


S4

S5

Average Ripple Rate (ripples/s)


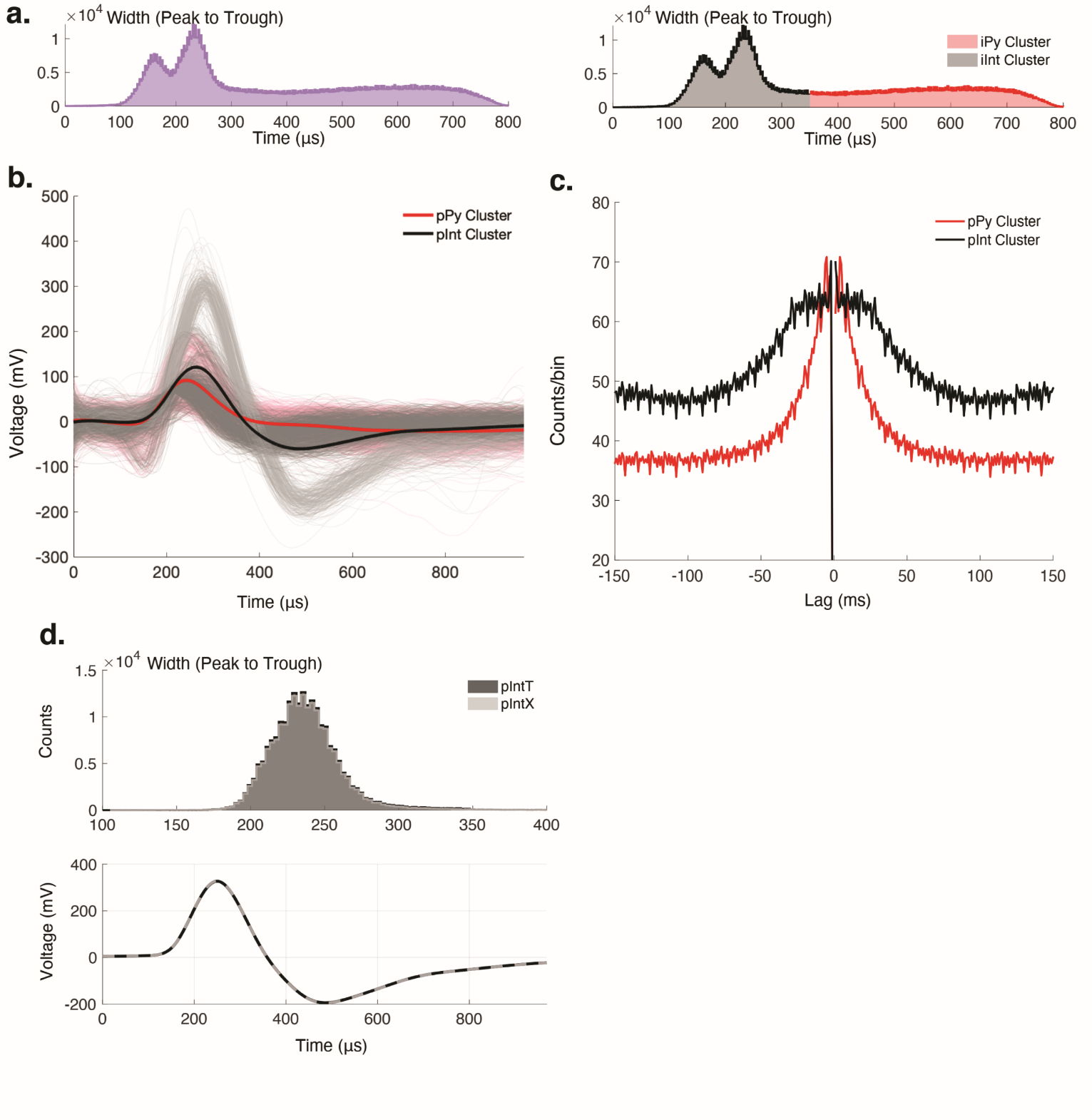


(µV)

Ground truth

pInt based on threshold

S6
